# The Application of Gail Model to Predict the Risk of Developing Breast Cancer among Jordanian Women

**DOI:** 10.1155/2020/9608910

**Published:** 2020-02-20

**Authors:** Hikmat Abdel-Razeq, Luna Zaru, Ahmed Badheeb, Shadi Hijjawi

**Affiliations:** ^1^Department of Internal Medicine, Section of Hematology and Medical Oncology, King Hussein Cancer Center, Amman, Jordan; ^2^School of Medicine, University of Jordan, Amman, Jordan; ^3^Intercept Pharmaceuticals, 4760 Eastgate Mall, San Diego, CA 9212, USA; ^4^King Khalid Hospital, P.O. Box: 1120, Najran, Saudi Arabia; ^5^CaroMont Health, 2525 Court Drive, Gastonia, NC 28054, USA

## Abstract

*Background and Objectives*. Breast cancer has been the most common cancer affecting women in Jordan. In the process of implementing breast cancer prevention and early detection programs, individualized risk assessment can add to the cost-effectiveness of such interventions. Gail model is a widely used tool to stratify patients into different risk categories. However, concerns about its applicability across different ethnic groups do exist. In this study, we report our experience with the application of a modified version of this model among Jordanian women.

**Methods:**

The Gail risk assessment model (RAM) was modified and used to calculate the 5-year and lifetime risk for breast cancer. Patients with known breast cancer were used to test this model. Medical records and hospital database were utilized to collect information on known risk factors. The mean calculated risk score for women tested was 0.65. This number, which corresponds to the Gail original score of 1.66, was used as a cutoff point to categorize patients as high risk.

**Results:**

A total of 1786 breast cancer patients with a mean age of 50 (range: 19–93) years were included. The modified version of the Gail RAM was applied on 1213 patients aged 35–59.9 years. The mean estimated risk for developing invasive breast cancer over the following five years was 0.54 (95% CI: 0.52, 0.56), and the lifetime risk was 3.42 (95% CI: 3.30, 3.53). Only 210 (17.3%) women had a risk score >0.65 and thus categorized as high risk. First-degree family history of breast cancer was identified among 120 (57.1%) patients in this high-risk group.

**Conclusions:**

Among a group of patients with an established diagnosis of breast cancer, a modified Gail risk assessment model would have been able to stratify only 17% into the high-risk category. The family history of breast cancer contributed the most to the risk score.

## 1. Introduction

**Figure 1:**
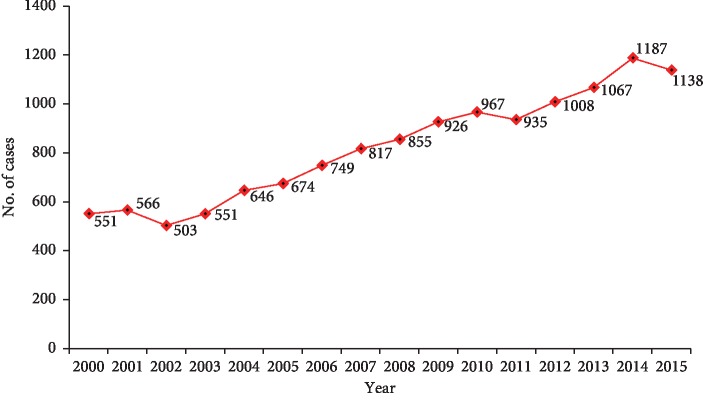
Breast cancer cases over years.

Breast cancer has been the most common cancer affecting women in Jordan [1]. While its incidence appears to be decreasing or leveling off in certain western countries [2, 3], incidence continues to rise in Jordan. A total of 1187 cases were reported in 2014 compared with only 445 in 1996 (the year when the Jordanian Cancer Registry was established) (Figure 1) [1, 4]. Compared with the West, and despite the recent establishment of early breast cancer detection programs, breast cancer is still diagnosed with more advanced stages where cure rates are low and cost of treatment is high. In addition, breast cancer affects the younger age group; the median age of affected Jordanian women is only 51 years, compared with 60 years in western societies [1, 5]. The national early detection program was recently established in Jordan, but with the current logistics and infrastructure, mass screening for breast cancer is not doable.

Individualized risk assessment for developing breast cancer is attracting more attention. The Gail model was developed to provide individualized breast cancer risk projection for American women, and it was the main tool for counseling high risk women and identifying women eligible for breast cancer prevention trials [6, 7]. The risk was calculated based on data from case control studies and SEER (surveillance, epidemiology, and end results) program data on age-specific breast cancer incidence and mortality rates for causes other than cancer. The application of the Gail model was tried on women with different ethnic groups, including African American women and reached different conclusions [8–10]. Establishment of risk assessment models can be more helpful in developing countries where the incidence of breast cancer is rising and the resources are limited to apply nationwide screening and early detection programs [11, 12]. The aim of this study is to find whether a modified version of the Gail model can identify a subgroup of Jordanian women at higher risk for developing breast cancer. Such high risk women can be the focus group for national screening and early detection programs.

## 2. Materials and Methods

### 2.1. Study Population

Women aged 18 years or older with histologically confirmed diagnosis of breast cancer were included. All women underwent a comprehensive breast cancer risk assessment in which information on known risk factors for breast cancer were collected. It includes demographics; family history of breast, ovarian, or any other cancer; reproductive history (age at menarche, age at first live birth, age at menopause, if applicable); history of benign breast disease (including number of biopsies), estrogen exposure (including oral contraceptive pills, hormone replacement therapy, or use of ovulation induction therapy).

A majority of the elements needed to calculate the risk of breast cancer using the Gail model were available in patients' medical records. Age used in our calculations was the age at diagnosis minus 5 years (assuming that the risk assessment was done 5 years prior to their breast cancer diagnosis). Deficient information was completed during patients' regular follow-up visits to our oncology clinics. The study was approved by our Institutional Review Board (IRB), and individual consent was exempted.

### 2.2. Statistical Analysis

The mean 5-year risk score and the mean life-time risk score were calculated using the SAS program downloaded from the NCI website. Patients were included in the analysis with the hypothetical assumption that they have not developed breast cancer.

The program was modified as follows: the average of Jordanian age-specific invasive breast cancer incidence rates over the years 2006–2010 was calculated and replaced the corresponding values in the original program. Similarly, age-specific mortality rates from other causes were replaced by the corresponding values published by the Ministry of Health [4]. Similar to what was originally done in the Gail model and breast cancer chemoprevention trials, the high-risk category was defined as those whose calculated risk for developing breast cancer is more than the calculated mean for women ≥60 years [6, 7]. All the analyses other than the summary of patients' characteristics were performed on a subgroup of patients aged between 35 and 59.9 years. Subgroup comparisons were carried out using the Chi-squared test for categorical variables and *t*-test or Wilcoxon rank test for continuous variables.

## 3. Results

Over period of five years, a total of 1786 breast cancer patients were treated and followed at our institution and included in this study. The mean age was 50 (range: 19–93) years. Patients were classified into three age groups; group I (<35 years): 182 (10.2%) patients, group II (35–59.9 years): 1213 (67.9%) patients, and group III (≥60 years): 391 (21.9%) patients.

Similar to the methodology used by the original Gail risk assessment model, the modified version was applied only on patients aged 35–59.9 (group II). Both younger and older groups were used for comparison. Patients' characteristics are summarized in Table 1.

Risk assessment was performed first for group III (age ≥60 years, 391 patients) utilizing the original Gail model; the mean (95% CI) risk for developing breast cancer in the next 5 years for this group was 1.66 (1.56–1.76). Using our modified model (with our local breast incidence and mortality rates), the calculated mean (95% CI) risk score for the same group was 0.65 (0.61–0.69). This score of 0.65, which corresponds to the original Gail score of 1.66, was used as a cutoff point to categorize patients as high risk.

Analysis was carried out on the 1213 study patients (aged 35–59.9 years). The mean (95% CI) estimated risk for developing invasive breast cancer over the coming five years, which will be referred to as modified Gail score, was 0.54 (0.52–0.56) and over lifetime was 3.42 (3.30–3.53). The application of the original Gail model in this group of patients would result in higher risk scores of 0.88 (0.85, 0.91) at 5 years and 9.22 (8.96–9.49) over lifetime. Among this group, only 210 (17.3%) women had a risk score >0.65 with a mean (95% CI) of 1.08 (1.01–1.15) and thus categorized as high risk.

Table 2 presents the Gail model factors that contributed to identifying the high-risk group. It details the differences between the high-risk and low-risk groups in mean age, age at menarche, and age at first live birth. Additionally, differences in family history of breast cancer and prior breast biopsies are also illustrated. Among the high-risk group, the first-degree family history of breast cancer was reported by 120 (57.1%), while prior breast biopsies were reported by 21 (10.0%). In this same high-risk group, the mean age (95% CI) at first pregnancy was 27.2 (26.3– 28.1) years.

The family history of “any” cancer in a first-degree relative was reported more (31.7%) in the eldest age group (≥60 years) compared to 20.3% in the youngest (<35 years) and 27.6% in our study group (35–59.9 years) (*p*=0.0198). The family history of “breast” cancer showed a similar trend with higher proportion (13.8%) identified in the eldest age group compared with 6.0% among the youngest and 11.8% in the study group (*p*=0.0253) (Table 3).

To further explore risk factors other than the first-degree family history of breast cancer, comparison between the group of patients with the first-degree family history of breast cancer and those without such history was performed among those considered at high risk with the modified Gail score >0.65. There was a difference in the mean age between the group with first-degree family history of breast cancer and the group without, with a corresponding mean (95% CI) of 48.6 (47.6– 49.6) and 51.2 (50.1– 52.3) years, respectively. Additionally, patients without the first-degree family history of breast cancer were significantly (*p* < 0.0001) older at their first live birth (mean (95% CI): 29.5 (28.5– 30.5)) than the group without family history (mean (95% CI): 24.6 (23.3–25.9)). The number of breast biopsies in patients without first-degree family history of breast cancer (14 (15.7%)) was significantly (*p*=0.018) higher than in the group with the first-degree family history of breast cancer (7 (5.8%)) (Table 4).

## 4. Discussion

Early detection and prevention of breast cancer is receiving more attention from healthcare planners especially in developing courtiers like ours [13]. For many reasons, many societies cannot apply mass screening programs for breast cancer; in such cases, defining and identifying group(s) of high-risk women for breast cancer is an attracting strategy [14, 15]. Different models were developed to perform a quantitative risk assessment [16]; the most famous model was the one developed by Gail et al. [7]. However, concerns about the applicability of this model across different nations and ethnic groups do exist [17, 18].

In this study, efforts were made to modify the original Gail model based on local data and vital statistics utilizing the same risk factors and calculation methods. Moreover, the threshold for risk classification (modified Gail score = 0.65) derivation was similar to the original Gail model and breast cancer chemoprevention trials. The mean “original” Gail score in the study group (patients 60 years or older) was 1.66 which concords the high-risk cutoff value in the original Gail model scoring system. Utilizing the modified model, only 17.3% of women with already established diagnosis of breast cancer would have been labeled as high risk 5 years prior to their diagnosis, thus missing more than 80% of women from inclusion into an early detection program or breast cancer chemoprevention trials. Our findings from this study highlight several other important points. First, accross all age groups, a high percentage of women (43%) reported a postive family history of cancer, 50% of them were breast cancer. When reviewing the subset of patients identified as high risk by the modified model, our data indicate that a positive family history of breast cancer is a major contributing factor to classify high risk group with 57% of them reported a positive first- or a second-degree relative with breast cancer. Second, on contrary to popular beliefs, such positive family history of cancer was commonly encountered in all age groups, not only younger patients. In fact, our data showed that a positive first-degree family history of any cancer, or “breast” cancer, was more frequently encountered in the older age group compared with the youngest one, 31.7% vs. 20.3% for any cancer and 13.8% vs. 6.0% for breast cancer. Third, in the absence of a family history of breast cancer, previous biopsies and older age at first birth contributed the most to high-risk scores.

Our finding of a very low percentage of breast cancer patients who underwent prior breast biopsies highlights the fact that few Jordanian women undergo routine breast clinical exam or mammography; the practice of either or both combined is expected to increase the number of women undergoing diagnostic breast biopsies.

Data presented in this report can be used by healthcare planners to direct breast cancer screening and early detection to the highest risk group possible. Applying the family history of breast cancer (1^st^ and 2^nd^ degree) would have picked most high-risk patients identified by the application of the full Gail model or its modified version.

In countries with limited resources, like ours, and while working on capacity building, healthcare planners can use a positive family history of breast cancer or age as criteria to enroll women in active screening programs [19]. The data presented in our study represent the first attempt to investigate breast cancer risk profiles in Jordanian women. There are many caveats in applying these findings, and it is anticipated that our findings will lead to further data collection to validate the presented results. The fact that we used patients diagnosed and treated at a single institution should not be a concern since our institution treats more than two-thirds of breast cancer patients in the country. However, enrolling all breast cancer at a national level will strengthen such conclusions.

In summary, the modified Gail model would have been able to detect only 17.3% of the population who were already diagnosed with breast cancer, if the score was calculated 5 years before their diagnosis. The family history of breast cancer was the main factor in the Gail model that contributed to identifying high-risk subjects. Therefore, the use of this scoring system in the early detection program in Jordan, even after modification reflecting the country's cancer registry and vital statistics data, is questionable. This brings up an imminent obligation to come up with a risk cancer score that address the factors contributing to developing breast cancer in Jordanian population.

Because breast cancer affects younger age group in Jordan and despite the continued debate over the value of screening mammography [20–23], the current recommendations established by the Jordanian Breast Cancer Program are to commence screening to women at age 40. Though our national registry data from 1996 to 2014 shows that the rate of breast cancer incidence among Jordanian women at all ages is lower than the corresponding rates in western societies, as the life expectancy of Jordanian women increases and differences in life style, late marriages, reproductive patterns, and environmental factors narrows, their risk of developing breast cancer also is likely to increase to approach a “western” level. We, and many other similar societies, should be ready and be prepared for this increasing number of breast cancer patients!

## Figures and Tables

**Table 1:** Patients' characteristics.

Characteristics		Number of patients	Mean	Median (range)

Age (years)	All	1786	50.0	48.0 (19–93)
	Group I (<35)	182	31.2	31.8 (16.1, 34.9)
	Group II (35–59.9)	1213	47.1	46.6 (35, 59.9)
	Group III (≥60)	391	67.0	65.7 (60, 93.5)

Age at menarche (year)	All	1243	13.2	13.0 (9.0, 18.0)
	Group II	879	13.1	13.0 (9.0, 18.0)

Age at first live birth (year)	All	852	23.2	23.0 (14.0, 41.0)
	Group II	599	23.5	23.0 (14.0, 41.0)

Age at menopause (year)	All	657	49.0	50 (30.0, 56.0)
	Group II	385	48.0	49(30.0, 56.0)

BMI^*∗*^	All	1648	30.3	29.7 (16.0, 61.6)
	Group II	1124	30.0	29.3 (16.6, 57.9)

Categorical variables:				

			All groups (*n* = 1786)	Group II (35–59.9)

Smoking history	Never		1629 (91.2%)	1089 (89%)
	Previous		70 (3.9%)	57 (4.7%)
	Current		87 (4.9%)	67 (5.5%)

Marital status	Single		147 (8.2%)	102 (8.4%)
	Married		1574 (88.1%)	1071 (88.3%)
	Unknown		65 (3.6%)	40 (3.3%)

History of benign breast biopsies	Yes		51 (2.9%)	35 (2.9%)
	No		1690 (94.6%)	1149 (94.7%)
	Unknown		45 (2.5%)	29 (2.4%)

^*∗*^BMI: body mass index.

**Table 2:** Characteristics of high- and low-risk patients according to the modified Gail model (age: 35–59.9 years)

Variable^*∗*^	High-risk group (modified Gail score >0.65) (*N* = 210)			Low-risk group (modified Gail score ≤0.65) (*N* = 1003)		
	*N*	Mean	95% CI	*N*	Mean	95% CI
Estimated risk for developing invasive breast cancer:						
(i) Over the next 5 years	210	1.08	(1.01, 1.15)	970	0.42	(0.41, 0.43)
(ii) Over lifetime (age 90)	210	5.9	(5.48, 6.33)	970	2.9	(2.8, 2.94)

Age	210	49.7	(48.9, 50.5)	1003	46.6	(46.1, 47.0)
Age at first pregnancy	163	27.2	(26.3, 28.1)	454	22.1	(21.7, 22.5)
Age at menarche	168	13.1	(12.9, 13.3)	711	13.1	(13.0, 13.2)
	*N*	Count (%)		*N*	Count (%)	

First-degree family with breast cancer	210	120 (57.1%)		1003	23 (2.3%)	
Prior breast biopsy	210	21 (10.0%)		975	14 (1.4%)	

**Table 3:** Family history of breast cancer by age group.

Family history	All ages (*n* = 1786)	Group I <35 years (*n* = 182)	Group II 35–59.9 years (*n* = 1213)	Group III ≥60 years (*n* = 391)	*p* value
Family history of any cancer:	760 (42.6%)	86 (47.2%)	520 (42.9%)	154(39.4%)	0.1948
(i) 1st degree	496(27.8%)	37 (20.3%)	335 (27.6%)	124 (31.7%)	0.0198
(ii) 2nd degree	401(22.5%)	58 (31.9%)	279 (23%)	64 (16.4%)	<0.001

Family history of breast cancer:	385(21.6%)	44 (24.2%)	260(21.4%)	81 (20.7%)	0.6337
(i) 1st degree	208 (11.6%)	11 (6.0%)	143 (11.8%)	54 (13.8%)	0.0253
(ii) 2nd degree	177 (9.9%)	33 (18.1%)	117 (9.6%)	27 (6.9%)	0.0040

The total of first degree and second degree does not match the presented total since some cases have both first- and second-degree family history of cancer/breast cancer.

**Table 4:** Comparison between patients with and without family history of breast cancer among high-risk patients (modified Gail score >0.65, age: 35–59.9; *n* = 210).

Variables	Patients with family history of breast cancer (*n* = 120)		Patients with no family history of breast cancer (*n* = 90)		*p* value
	Mean	95% CI	Mean	95% CI	
Age	48.6	(47.6, 49.6)	51.2	(50.1, 52.3)	<.0001
Age at first pregnancy	24.6	(23.3, 25.9)	29.5	(28.5, 30.5)	
Age at menarche	13.2	(12.9, 13.4)	12.9	(12.6, 13.2)	

Prior breast biopsy	Count (%)		Count (%)		0.018
	7 (5.8%)		14 (15.7%)		

## Data Availability

The data used to support the findings of this study are available from the corresponding author upon request.
